# A Segment of 97 Amino Acids within the Translocation Domain of *Clostridium difficile* Toxin B Is Essential for Toxicity

**DOI:** 10.1371/journal.pone.0058634

**Published:** 2013-03-06

**Authors:** Yongrong Zhang, Lianfa Shi, Shan Li, Zhiyong Yang, Clive Standley, Zhong Yang, Ronghua ZhuGe, Tor Savidge, Xiaoning Wang, Hanping Feng

**Affiliations:** 1 School of Bioengineering, East China University of Science and Technology, Shanghai, China; 2 Department of Microbial Pathogenesis, University of Maryland Dental School, Baltimore, Maryland, United States of America; 3 University of Massachusetts Medical School, Worcester, Massachusetts, United States of America; 4 Baylor College of Medicine, Houston, Texas, United States of America; University of Helsinki, Finland

## Abstract

*Clostridium difficile* toxin B (TcdB) intoxicates target cells by glucosylating Rho GTPases. TcdB (269 kDa) consists of at least 4 functional domains including a glucosyltransferase domain (GTD), a cysteine protease domain (CPD), a translocation domain (TD), and a receptor binding domain (RBD). The function and molecular mode of action of the TD, which is the largest segment of TcdB and comprises nearly 50% of the protein, remain largely unknown. Here we show that a 97-amino-acid segment (AA1756 – 1852, designated as ?97 or D97), located in the C-terminus of the TD and adjacent to the RBD, is essential for the cellular activity of TcdB. Deletion of this segment in TcdB (designated as TxB-D97), did not adversely alter toxin enzymatic activities or its cellular binding and uptake capacity. TxB-D97 bound to and entered cells in a manner similar to TcdB holotoxin. Both wild type and mutant toxins released their GTDs similarly in the presence of inositol hexakisphosphate (InsP_6_), and showed a similar glucosyltransferase activity in a cell-free glucosylating assay. Despite these similarities, the cytotoxic activity of TxB-D97 was reduced by more than 5 logs compared to wild type toxin, supported by the inability of TxB-D97 to glucosylate Rac1 of target cells. Moreover, the mutant toxin failed to elicit tumor necrosis factor alpha (TNF-α) in macrophages, a process dependent on the glucosyltransferase activity of the toxin. Cellular fractionation of toxin-exposed cells revealed that TxB-D97 was unable to efficiently release the GTD into cytosol. Thereby, we conclude the 97-amino-acid region of the TD C-terminus of TcdB adjacent to the RBD, is essential for the toxicity of TcdB.

## Introduction

*Clostridium difficile* exotoxins TcdA and TcdB are the primary virulence factors of *C. difficile* infection (CDI). Both toxins belong to a family of large clostridial toxins that glucosylate small Rho GTPases [1], [2]. TcdB is generally more cytotoxic to cultured cells than TcdA [3], but exhibits little enterotoxicity in animal models [4], [5], [6]. However, recent studies have shown that isogenic *C. difficile* strains expressing only functional TcdB also cause CDI in hamsters [7], [8]. The mechanism for these apparently discordant observations is currently unknown, but may relate to TcdB being produced locally in the caecum by *C. difficile* in the presence of co-accessory virulence factors [9].

TcdB is a multi-domain toxin consisting of four distinct functional domains: an N-terminus glucosyltransferase domain (GTD), a cysteine protease domain (CPD), a translocation domain (TD), and a C-terminus receptor binding domain (RBD, also known as combined repetitive oligopeptides or CROP) [10], [11], [12]. The molecular mode of action of the toxin is not completely understood but a model can be built based on current data. The toxin is thought to bind to cells and subsequently to be endocytosed. Acidification of endosome leads to a conformational change of the toxin, exposing the hydrophobic region within the TD to inserts into the endosomal membrane [13]. The TD may form a pore permitting the transport of the GTD and CPD into the cytosol [14], [15]. The CPD may then be activated by allosteric binding of cyotosolic inositol *hexakis*phosphate (InsP_6_), resulting in toxin self-cleavage and release of the GTD into cytosol [16], [17], [18], although *in vivo* studies are necessary to confirm this mechanism of action. The GTD, a glucosyltransferase, inactivates Rho GTPases including RhoA, Rac1, and CDC42, by covalently linking a glucose moiety onto them [1], [2]. TD, the largest segment of the full length TcdB, is thought to transport the GTD into the cytosol from the endosome. Knowledge of how exactly this process occurs and which regions of the TD are critical for this process is starting to emerge [15]. In this study, we identified a small segment of 97 amino acids (AA1756 – 1852, designated as D97), located in the C-terminus of TD of TcdB that is essential for its toxicity.

## Materials and Methods

### Generation of TxB-D97

To generate TxB-D97, the gene encoding RBD and a 97-amino-acid segment (D97) of C-terminus of TD was deleted. We then re-installed the sequence of the full-length of RBD (from amino acid 1852 to 2336) using PCR. Primers used in PCR reaction were 5′- AAT TAC TAG TAT AAC CGG TTT TGT GAC TGT AGG CGA TG-3′ sense, 5′- ATA TCC CGG GTT AGT GAT GGT GAT GGT GAT GCG ATC C-3′ anti-sense. TcdB and TxB-D97were expressed and the *B. megaterium* bacteria were lysed with One-shot Cell Disruptor (Constant System Limited, GA). The recombinant proteins were purified as previously described [19].

### Cytotoxic and cytopathic effects of TxB-D97 on cultured cells

Cytotoxicity of the toxins on cultured cells was assessed by MTT ((3-(4,5-Dimethylthiazol-2-yl)-2,5-diphenyltetrazolium bromide) viability assay as described previously [20]. Cytopathic effects of the toxins on cultured cells were assessed by cell rounding assays. The African green monkey kidney cell line Vero cells seeded in 96-well plates were treated with wild type and mutant TcdB. Cell rounding was visualized by phase-contrast microscopy. About 100 cells from 5 random fields were counted and the percentage of rounded cells was determined [20]. The experiments were repeated three times, and triplicate wells were assessed for cell rounding in each experiment.

### Glucosyltransferase activity of the toxins

GT activity of TcdB and TxB-D97 was measured by their ability to glucosylate Rho GTPase Rac1 in cell lysates. Vero cell pellets were resuspended in a reaction buffer (50 mM HEPES, pH 7.5, 100 mM KCl, 1 mM MnCl_2_ and 2 mM MgCl_2_) and lysed by passing through a 30 G needle for 40 times. After centrifugation (167,000 g, 3 min), the supernatant was used as a cytosolic fraction (protein concentration between 2-5 mg/ml). To perform the glucosylation assay, the cytosolic fraction was incubated with TcdB or TxB-D97 at different concentrations at 37 °C for 30 min. The reaction was terminated by adding SDS-sample buffer and samples were heated at 100 °C for 5 min before loading on a 12% SDS-PAGE gel. An antibody that specifically recognizes the non-glucosylated form of Rac1 (clone 102, BD Bioscience), anti-β-actin (clone AC-40, Sigma), and HRP-conjugated anti-mouse-IgG (Amersham Biosciences) were used for detection using standard western blotting analysis.

### Fluorochrome labeling of wild type and mutant toxins

The fluorochrome labeling was carried out as described previously [21]. TcdB and TxB-D97 proteins (1 mg/ml) were labeled with Alexa Fluor 488 following the manufacturer’s instructions (Invitrogen). Free fluorescence dyes were removed by passing through a desalting column twice by centrifugation. The labeled toxins had a similar molar ratio of Alexa-488 to the proteins at 1.24 for TcdB and at 1.18 for TxB-D97. The activity of the labeled wild type toxins remained unchanged as measured by cytotoxicity assay (data not shown).

### Analysis of binding and internalization of toxins

Vero cells were exposed to the fluorochrome-labeled toxins at 10 µg/ml at 37 °C, or at 10, 30 µg/ml at 4 °C, for 30 min followed by flow cytometry analysis as described previously [22]. Further increasing toxin concentration up to 50 µg/ml did not enhance the binding. For imaging analysis, cells on coverslips were exposed to the fluorochrome-labeled toxins for 30 min either at 37 °C or on ice. The cells were washed and fixed with formaldehyde before being visualized under a laser-scanning microscope as described previously [23]. Briefly, Coherent Innova 70C lasers were used (argon ion and an argon-krypton ion) to produce the 488 and 568 nm excitation, respectively. Images were obtained through a Plan APO 60× objective with a numerical aperture of 1.45 and were digitally recorded (640×448-pixel) by a charge-coupled device camera developed by Lincoln Laboratory (MIT). A Physik Instruments PIFOC was used for fine focus control. The effective pixel size at the specimen is 160 nm in x-y and 250 nm in z direction, and a 3D stack of 40 image planes was acquired for each cell. Fluorescence images were deconvolved with a constrained, iterative approach originally designed for UNIX systems [24]. The algorithm was rewritten using FFTW, a free, fast Fourier transform library and implemented as a multiuser client/server system on computers running the Fedora operating system (Red Hat, Durham, NC), either as a stand-alone or configured in a Beowulf cluster. Each image was dark current and background subtracted, flat-field corrected, and then deconvolved. The deconvolution images were thresholded to eliminate non-specific binding. Voxels that fell below a threshold were considered to be non-specific bindings and were set to zero; all other voxels remained unchanged. This threshold was derived from analysis of control images of cells exposed to unlabeled toxins. The intensity which eliminates 95% of voxels in the control images becomes the threshold intensity. Interactive three-dimensional visualization was performed using Data Analysis and Visualization Environment software [25].

### InsP_6_-induced autocleavage of the toxins

The InsP_6_-induced autocleavage of the toxins was carried out as described previously [16]. All toxin proteins were diluted in 10 mM Tris (pH 7.5) buffer to a concentration of 10 µg/ml in a final volume of 20 µl. The reaction was initiated by addition of InsP_6_ (10 µM) in absence of DTT at the indicated final pH values or incubated for the indicated time. For some experiments, the toxins were incubated at room temperature overnight. The reactions were stopped by SDS sampling buffer and analyzed by Western blot using alpaca anti-TcdB polyclonal antibodies (generated in this laboratory).

### TNS fluorescence analysis of TcdB and TxB-D97

pH-induced conformational change of the toxins was examined as described previously [21]. Briefly, 2-(*p*-Toluidinyl) naphthalene-6-sulfonic acid, sodium salt (TNS, Invitrogen) solutions were prepared in pH 4.0 or pH 7.4 buffer (100 mM NaCl, 100 mM ammonium acetate, 1 mM EDTA). TNS was added to each buffer to a final concentration of 150 µM. TcdB or TxB-D97 (5 µg) was mixed into 500 µl of the pH buffer. Mixtures were incubated at 37 °C for 20 min. In some experiments, the buffer pH containing the toxins was slowly neutralized from pH 4 to pH 7.4 to assess refolding of the toxins. Each sample was analyzed on a SLM 8100 photon-counting fluorometer (Spectronic Instruments, Rochester, N.Y.) with an excitation of 366 nm and an emission scan of 380 to 500 nm.

### Intracellular cytokine staining

RAW 264.7 cells were exposed to 1 ng/ml TcdB or 100 ng/ml TxB-D97 for 6 hr. Cells were harvested and TNF-α production was determined by intracellular cytokine staining as described previously [26].

### Detection of GTD in the cytosolic faction of toxin-exposed cells by immunopecipitation

Vero cells growing on 10-cm dishes were exposed to 1 µg/ml of TcdB or TxB-D97 for 2 hr at 37°C. Culture media were removed and cells were rinsed with ice-cold PBS 3 times before harvesting. Cells were collected in lysis buffer containing 250 mM sucrose, 100 mM KCl, 10 mM N-ethylmaleimide and 1 mM PMSF and lysed by passing through a syringe (30G, 40 passes through the needle) before centrifugation at 100,000 g for 1 hr to separate cytosolic fractions. The GTD in the cytosolic fraction was precipitated using biotinylated VHH monoclonal antibody (clone E3 (∼ 35 kDa) generated in our laboratory) against GTD of TcdB and streptavidin beads. The presence of GTD in the pull-down beads was measured by immunoblotting with VHH monoclonal antibody (clone C6 generated in our laboratory) against GTD of TcdB and HRP-conjugated goat anti-alpaca secondary antibody (Bethyl Laboratories, TX).

### Mouse systemic toxin challenge

Six- to 8-week-old CD1 mice were purchased from Jackson Laboratory (MI, USA). Mice were housed in dedicated pathogen-free facilities. The animal study was carried out in strict accordance with the recommendations in the Guide for the Care and Use of Laboratory Animals of the National Institutes of Health. The protocol was approved by the Committee on the Ethics of Animal Experiments of the University of Maryland Dental School (protocol #12-03-01). Mice were sacrificed when became moribund after toxin challenge, and all efforts were made to minimize suffering. To assess *in vivo* toxicity of the mutant toxin, groups of mice were challenged intraperitoneally with either lethal dose (LD_100_) of wild type TcdB (100 ng/mouse) or 100 µg/mouse TxB-D97 (1000x LD_100_ of TcdB). Mouse survival was monitored and data were analyzed by Kaplan–Meier survival analysis with Logrank test of significance.

## Results

### Generation of TxB-D97

**Figure 1:**
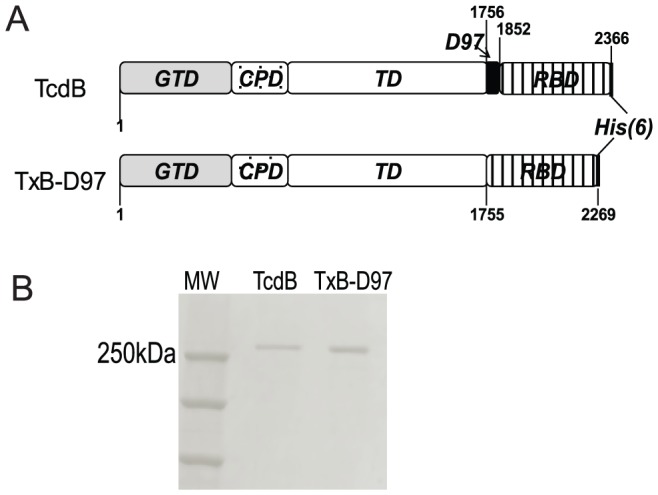
Size and Structure of Toxins. (A) Domain structure of TcdB and TxB-D97. GTD: glucosyltransferase domain; CPD: cysteine protease domain; TD: translocation domain; RBD: receptor binding domain, the region deleted in TxB-D97 is shown in black, His(6): 6-histidine tag. (B) Coomassie stained SDS-polyacrylamide gel showing purified toxins.

Diagrams of TcdB and TxB-D97 are shown in Figure 1A. The deleted segment designated as D97 (AA1756–1852) is located at the C-terminal end of translocation domain (TD), adjacent to the receptor binding domain (RBD, also known as CROP). TxB-D97 was generated by deletion of gene encoding D97 and RBD of TcdB followed by reinstalling the full-length RBD. Purification of TcdB and TxB-D97 resulted in a single band on Coomassie blue stained SDS polyacrylamide gels, sized at approximately 269 kDa for TcdB and 260 kDa for TxB-D97 (Figure 1B).

### TxB-D97 essentially loses its toxicity

**Figure 2:**
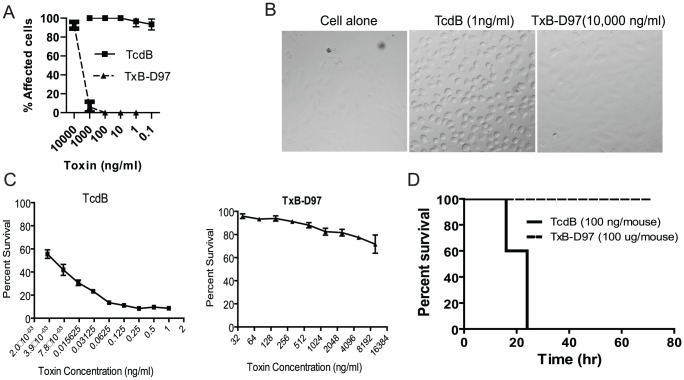
*In vitro* and *in vivo* toxicity of TcdB and TxB-D97. Vero cells were incubated with different concentrations of TcdB and TxB-D97 for 24 hr (A) or 4 hr (B). The percentage of cell rounding was determined by microscopy. (C) Vero cells were exposed to the indicated amount of TcdB and TxB-D97 for 3 days. The MTT assay was performed and cell viability was expressed as the percentage of survival cells without exposure to toxins. (D) Groups of mice were ip injected with 100 ng of TcdB or 100 µg of TxB-D97. Mouse survival was monitored and data were analyzed by Kaplan–Meier survival curve with Logrank test of significance. (n  = 5, P  =  0.0007).

TcdB is a potent cytotoxin to most mammalian cells causing disruption of cytoskeleton [27]. Deletion of D97 dramatically attenuated this activity**.** As shown in Figure 2A, TcdB induced cell rounding in over 90% of Vero cells at a concentration of 0.1 ng/ml after 24 hr, while TxB-D97 only induced a similar rate of cell rounding at a concentration of 10,000 ng/ml. Additionally, TcdB caused 100% of cells rounding at the concentration of 1 ng/ml after 4 hr of incubation whereas TxB-D97-exposed cells showed no observable change even at a concentration of 10,000 ng/ml (Figure 2B). MTT viability assay showed that TcdB exhibited a significant cytotoxicity to Vero cells in pg/ml range whereas TxB-D97 started to show toxicity in µg/ml of doses after 3 days of toxin incubation (Figure 2C). Therefore, the cytotoxicity of TxB-D97 was reduced by more than 5 logs as compared to wild type TcdB, indicating that D97 is critical for cytotoxicity in cultured cells. Finally, we assessed *in vivo* toxicity of the TxB-D97 by challenging mice systemically. Mice challenged with 100 ng of TcdB developed signs of systemic disease rapidly and all died or became moribund within 24 hr (Figure 2D). On the contrary, none of TxB-D97-injected mice (at dose of 1000 fold of LD_100_) exhibited any sign of disease (Figure 2D). This data clearly demonstrate that the deletion of D97 essentially abolishes the *in vivo* toxicity of TcdB.

### Cellular binding and uptake of TxB-D97 is similar to TcdB

**Figure 3:**
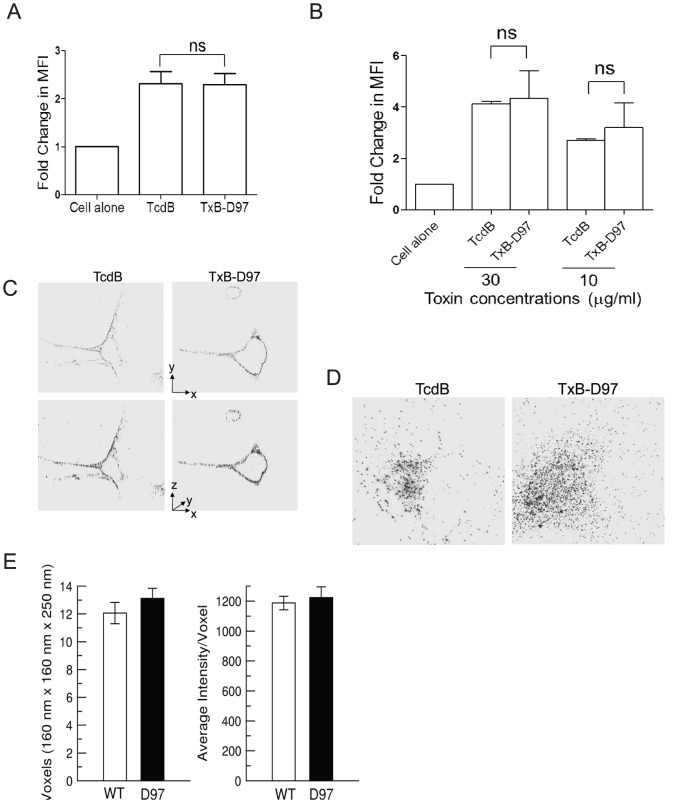
Binding and internalization of the toxins. Vero cells were exposed to Alex-488 labeled TcdB or TxB-D97 for 30 min at 37 °C (A, D) or on ice (B, C) before harvesting for flow cytometry (A, B) or laser-scanning microscopy (C, D) analysis. (A, B) Vero cells were exposed to 10 µg/ml in (A) or the indicated amounts (B) of the toxins. Mean fluorescent intensity (MFI) of the cells was measured by flow cytometry analysis and data show fold changes of MFI of toxin-exposed cells over untreated control (ns  =  not significant). (C) The images reveal the localization of TcdB (left) and TxB-D97 (right) in a section of 1 μm thickness through the middle of the cells. The same images are viewed from two different angles in the upper and lower panels as indicated by the vectors on the right. Note that both forms of TcdB are located on the surface. (D) Images show the examples of cellular distribution of TcdB (left) and TxB-D97 (right) after their internalization in a section of 1 µm thickness through the middle of the cells. (E) The size (left) and fluorescence intensity (right) of toxin-containing vesicles in (D) are quantified. Data represent as mean ± SEM. N =  9 for TcdB and 11 for TxB-D97; P = 0.321 TcdB size vs TxB-D97 size; P  = 0.064 fluorescent intensity TcdB-exposed cells vs. those of TxB-D97.

**Figure 4:**
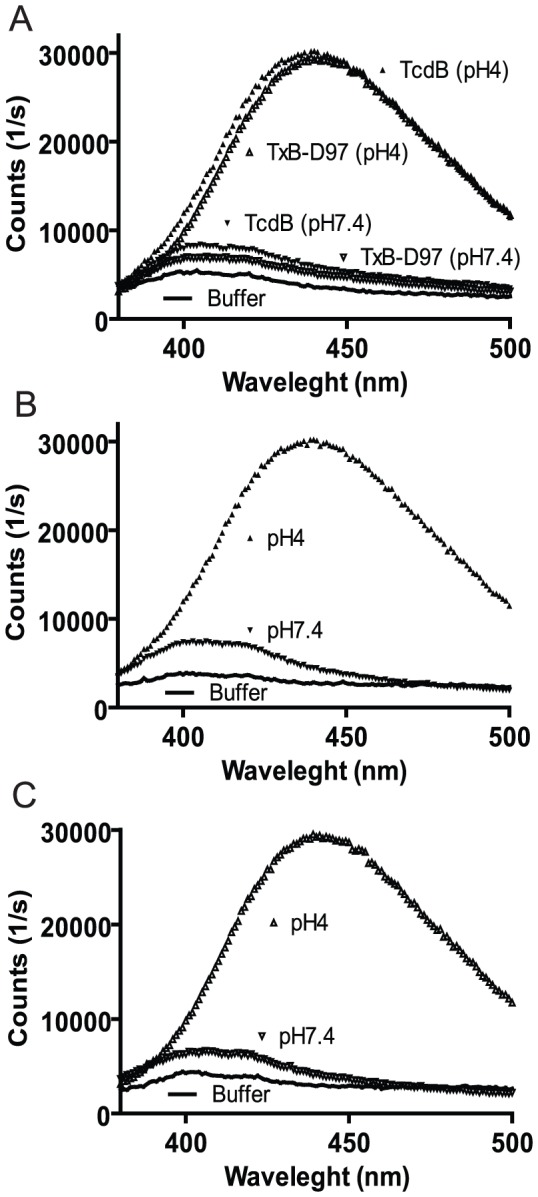
pH-induced conformational change of the toxins. (A) Five µg of either TcdB or TxB-D97 were diluted into 500 µl of pH 4.0 and 7.4 buffer before adding 2,6-TNS at a final concentration of 150 µM. (B, C) The pH of buffer containing TcdB (B) or TxB-D97 (C) was neutralized from pH 4 to pH 7.4. The binding of 2, 6-TNS to hydrophobic region of the proteins led to an increase of fluorescence measured under a photon-counting fluorometer.

The RBDs (or CROPs) of the *C. difficile* toxins were implied to function as the receptor binding domain [27]. Although TxB-D97 has the same RBD as wild type TcdB, we examined whether the binding and uptake of TcdB is affected by the deletion of D97. Vero cells were exposed to fluorochrome-labeled toxins, and flow cytometry showed similar levels of fluorescence intensity in cells exposed to wild type TcdB or TxB-D97 either at 37 °C (Figure 3A) or 4 °C (Figure 3B) (determined by the average fold changes in mean fluorescent intensity (MFI) of the toxin-exposed cells over untreated control cells). The toxins did not exhibit a saturated surface binding at the dose of 10 µg/ml (Figure 3B). We therefore further compared the cell surface binding and uptake of the toxins at this dose using quantitative imaging analysis. When cells were exposed to the toxins on ice, the surface membrane binding of TxB-D97 was similar to that of TcdB (Figure 3C). At 37 °C, both toxins were internalized similarly and there were no significant differences in toxin cellular distribution, or in the size and intensity of toxin-associated vesicles between the wild type and mutant toxins (Figure 3D and E). These data indicate that deletion of D97 does not interfere with toxin binding or uptake by cells.

### The response of TcdB and TxB-D97 to pH-induced conformational changes

TcdB undergoes structural changes under acidic conditions [21], which are believed to be necessary for membrane insertion of the toxin. We examined whether the D97 deletion affected TcdB conformational changes under acidic conditions using TNS as a probe as described previously [21]. TNS-associated fluorescence of both wild type and mutant toxins increased dramatically when toxins were incubated in pH 4.0 buffers (Figure 4A), suggesting that TxB-D97 also underwent conformational changes following acidification. Additionally, when the pH of the buffers was adjusted back to 7.4, the fluorescence intensity of TxB-D97 was reduced to a level close to buffer control (Figure 4B and C). When comparing side by side to wild type TcdB, the fluorescent intensity of TxB-D97 at both neutral and acidic pH was slighter lower than that of TcdB, but the difference may not be significant. Nonetheless, the data suggest that the deletion of D97 may alter the structure of the translocation domain of TcdB.

### Cysteine protease and glucosyltransferase activities of wild type and mutant toxins

**Figure 5:**
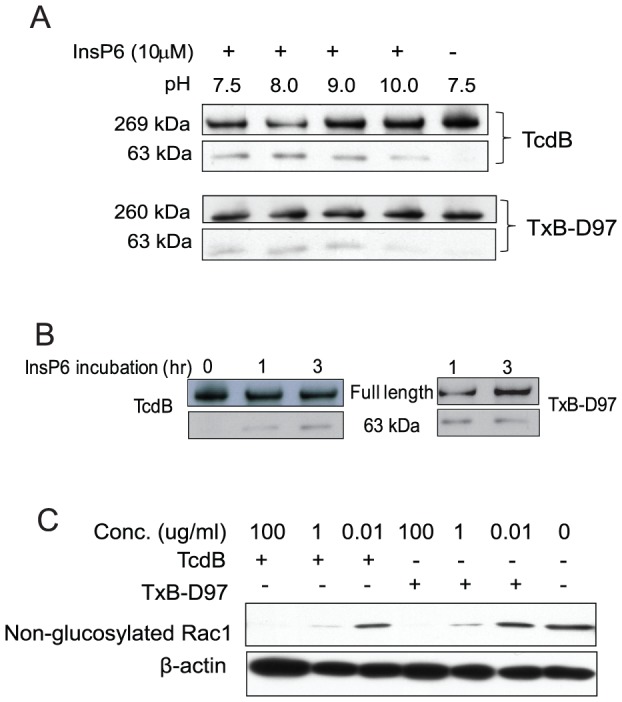
Enzymatic activities of TcdB and TxB-D97. (A, B) TcdB or TxB-D97 (10 ng/ml) was incubated with 10 µM InsP_6_ in different pH buffers at RT for 24 hr (A), or in neutral pH buffer at RT for the indicated time (B). The autocleavage and release of GTD (63 kDa) was examined by western-blotting using alpaca anti-TcdB polysera. (C) Vero cells were lysed and the cytosolic fraction was exposed to the toxins at different concentrations for 1 hr before analyzed by immunoblotting using monoclonal antibody (Clone 102) that only binds to non-glucosylated Rac1. β-actin was used as an equal loading control.

Next we examined whether deletion of D97 affects the toxin cysteine protease and glucosyltransferase activities. TxB-D97 and TcdB were incubated with InsP_6_ at various pH buffers and the release of GTD was detected by western blotting. Figure 5A shows that the pattern of InsP_6_-induced autocleavage and release of the 63-kDa of GTDs was similar between TxB-D97 and wild type TcdB. Moreover, shorter incubation times of the toxins with InsP_6_ also yielded a similar autocleavage and release of the GTDs between the wild type and the mutant (Figure 5B). Next, we assessed the glucosyltransferase activity of the wild type and mutant toxins using Vero cell lysate as a substrate and glucosylation of Rho GTPase Rac1 was measured. Both TcdB and TxB-D97 induced a similar dose-dependent Rac1 glucosylation (Figure 5C). These data suggest that deletion of D97 does not affect the intrinsic enzymatic activities of either cysteine protease or glucosyltransferase of the toxin.

### Release of GTD into the cytosol of host cells

**Figure 6:**
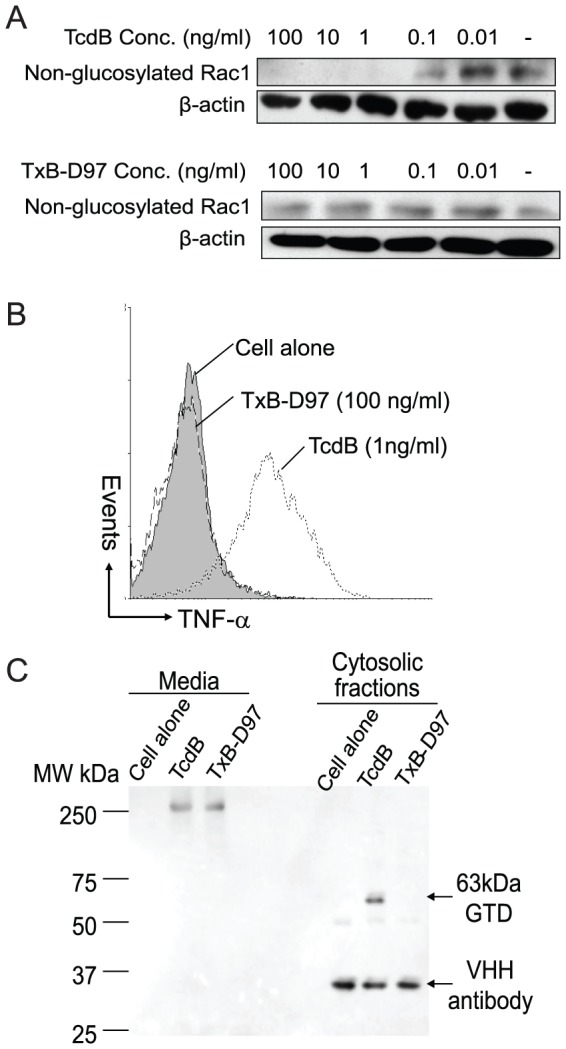
Cytosol delivery of GTD of the toxins. (A) Vero cells were exposed to different doses of TcdB or TxB-D97 for 4 hr. Cells were harvested and western blot was performed using monoclonal antibody (Clone 102) that only binds to non-glucosylated Rac1. β-actin was used as an equal loading control. (B) RAW264.7 cells were treated with the toxins at the indicated doses for 6 hr. TNF-α was measured by an intracellular cytokine staining followed by flow cytometry analysis. (C) Vero cells were exposed to TcdB or TxB-D97 (1 µg/ml) at 37°C for 2 hr. cells were lysed and GTD from the cytosol fractions was pulled down with VHH antibody against GTD of TcdB followed by western blotting analysis.

To gain insights of potential role of D97 region in the intoxication process of host cells, we performed a series of experiments. First, we examined whether TxB-D97 exposure of intact cells would lead to glucosylation of Rho GTPases. After cells were exposed to different concentrations of the toxins, TxB-D97 treatment failed to induce glucosylation of Rac1 at the concentrations tested, whereas TcdB caused a dose-dependent Rac1 glucosylation (Figure 6A). Second, we investigated whether TxB-D97 was able to induce TNF-α production in murine macrophages, an outcome dependent upon cytosolic glucosyltransferase activity of *C. difficile* toxins [26]. Figure 6B shows that TxB-D97 was unable to induce TNF-α expression in RAW 264.7 cells, even at a concentration 100 times higher than wild type TcdB. Finally, we directly measured the presence of GTD in the cytosolic fraction of toxin-treated cultured cells. After 2 hr of exposure of wild type or mutant toxins, Vero cells were lysed and cytosolic fractions were separated for immunoprecipitation to pull down GTDs in cytosolic fractions. Western blotting showed that a 63 kDa of GTD fragment was detected in the cytosol from cells treated with TcdB but not TxB-D97 (Figure 6C), whereas similar amounts of full-length wild type or mutant toxins remained in the culture media (Figure 6C). In summary, these data indicate that TxB-D97 is unable to efficiently deliver its GTD into the cytosol of intoxicated cells.

## Discussion

Bacterial protein toxins have evolved elegant machineries to deliver effector domains into host cells. *Clostridium difficile* toxin B has several domains that are involving in the delivery of its effector GTD into target cells: a RBD that presumably binds to host receptors followed by endocytosis of the toxins; a TD that undergoes conformational changes in the acidic endosome and inserts into the endosomal membrane, leading to the translocation of N-terminus into the cytosolic side of endosome; and a CPD that is activated by cytosolic InsP_6_ and self-cleaves the toxin to release the GTD into the cytosol. Thus, the intact domain functions are essential for the cytosolic delivery of GTD and for intoxication of host cells. In this study, we found that the deletion of a 97 amino-acid segment (D97), located in the C-terminus of TD markedly reduces the toxic activity of TcdB. Therefore, this segment appears to be a crucial structure for the function of other domain(s) in TcdB.

To identify the domain(s) affected by deletion of D97 and to understand the mechanism underlying the diminished cytotoxicity of TxB-D97, we examined the individual domain functions of TxB-D97. Since D97 is located at the RBD N-terminus of TcdB, we quantitatively examined whether its deletion affected the binding and uptake of the mutant toxin. Both flow cytometry analysis of total toxin-exposed cells and imaging analysis of representative individual cells showed a similar level of binding and uptake of TxB-D97 compared with wild type TcdB. The pattern of cellular distribution and the average sizes of toxin-associated vesicles between the TcdB and TxB-D97 exposed cells were also comparable. These data therefore indicated that the deletion of D97 does not affect the cellular binding or uptake of the toxin. We further found that D97 does not affect the enzymatic functions of the toxin, since the wild type TcdB and mutant TxB-D97 have comparable cysteine protease and glucosyltransferase activities.

The observation that TxB-D97 is essentially lost for its cytotoxic activity in cultured cells, yet maintains its cellular binding and internalization capacity, cysteine protease and glucosyltransferase activities, suggests that D97 may have some role in translocating the GTD across the endosomal membrane into the cytosol. Our results support this notion: 1) Exposure of cells with TcdB, but not TxB-D97, led to the glucosylation of Rho GTPase Rac1; 2) Unlike wild type TcdB, TxB-D97 failed to induce TNF-α expression in murine macrophages, an outcome dependent on cytosolic glucosyltransferase activity [26]; and 3) GTD fragment was detected in the cytosolic fraction from TcdB, but not from TxB-D97 exposed cells. Therefore, although TxB-D97 is endocytosed as efficiently as wild type TcdB into cells, its GTD is most likely trapped inside the endosome. The mechanism of action of the D97 segment in this virulence process is currently unknown. According to the 3D structural model of TcdB proposed by Pruitt and coworkers [28], the D97 is most likely located in a hinge region between RBD and TD, but is spatially close to the GTD. Therefore, deletion of the D97 segment may interfere with the recruitment of the GTD towards the membrane transporting apparatus of the TD, thus limiting GTD translocation across the membrane. Further experiments are necessary to test this hypothesis.

Recently Genisyuerek *et al* studied sequence elements within the TD of TcdB that are important for the pore-forming function [15]. In this study, a chimera TcdB_1-1755_-DTRD toxin was created by replacing C-terminal part (including the D97 and RBD of TcdB) with the receptor binding domain of diphtheria toxin (DTRD). TcdB_1-1755_-DTRD still retained cytotoxicity, but this was reduced by at least 4–5 logs as compared with wild type TcdB [15]. Although this result is in agreement with our findings, it is difficult to make a direct comparison since the efficiency of receptor binding and uptake of this chimeric toxin may be different from TxB-D97 due to the different receptor binding domains. Nonetheless, Genisyuerek *et al* hypothesized that the region from 1500 to 1850 might harbor a secondary receptor binding site. If this is correct, the additional receptor-binding site be located in the upstream region of D97 since our data suggest that D97 does not affect receptor binding of TcdB to host cells.

In summary, D97 of TcdB, located in the C-terminus of TD and adjacent to RBD, is critically important for the toxin activities. Targeted deletion of this region may allow us to generate suitable vaccine candidates against *C. difficile* infection.

## References

[1] Just I, Fritz G, Aktories K, Giry M, Popoff MR, et al (1994) Clostridium difficile toxin B acts on the GTP-binding protein Rho. J Biol Chem 269: 10706–10712. 8144660

[2] Just I, Selzer J, Wilm M, von Eichel-Streiber C, Mann M, et al (1995) Glucosylation of Rho proteins by *Clostridium difficile* toxin B. . Nature 375: 500–503. 7777059 10.1038/375500a0

[3] Chaves-Olarte E, Weidmann M, Eichel-Streiber C, Thelestam M (1997) Toxins A and B from Clostridium difficile differ with respect to enzymatic potencies, cellular substrate specificities, and surface binding to cultured cells. J Clin Invest 100: 1734–1741. 9312171 10.1172/JCI119698 PMC508356

[4] Mitchell TJ, Ketley JM, Haslam SC, Stephen J, Burdon DW, et al (1986) Effect of toxin A and B of Clostridium difficile on rabbit ileum and colon. Gut 27: 78–85. 3949240 10.1136/gut.27.1.78 PMC1433160

[5] Lyerly DM, Saum KE, MacDonald DK, Wilkins TD (1985) Effects of Clostridium difficile toxins given intragastrically to animals. Infect Immun 47: 349–352. 3917975 10.1128/iai.47.2.349-352.1985 PMC263173

[6] Lima AA, Lyerly DM, Wilkins TD, Innes DJ, Guerrant RL (1988) Effects of Clostridium difficile toxins A and B in rabbit small and large intestine in vivo and on cultured cells in vitro. Infect Immun 56: 582–588. 3343050 10.1128/iai.56.3.582-588.1988 PMC259330

[7] Lyras D, O'Connor JR, Howarth PM, Sambol SP, Carter GP, et al (2009) Toxin B is essential for virulence of *Clostridium difficile* . Nature 458: 1176–1179. 19252482 10.1038/nature07822 PMC2679968

[8] Kuehne SA, Cartman ST, Heap JT, Kelly ML, Cockayne A, et al (2010) The role of toxin A and toxin B in Clostridium difficile infection. Nature 467: 711–713. 20844489 10.1038/nature09397

[9] Carter GP, Rood JI, Lyras D (2010) The role of toxin A and toxin B in Clostridium difficile-associated disease: Past and present perspectives. Gut Microbes 1: 58–64. 20664812 10.4161/gmic.1.1.10768 PMC2906822

[10] Jank T, Aktories K (2008) Structure and mode of action of clostridial glucosylating toxins: the ABCD model. Trends Microbiol 16: 222–229. 18394902 10.1016/j.tim.2008.01.011

[11] Jank T, Giesemann T, Aktories K (2007) Rho-glucosylating Clostridium difficile toxins A and B: new insights into structure and function. Glycobiology 17: 15R–22R. 10.1093/glycob/cwm004 17237138

[12] Just I, Gerhard R (2004) Large clostridial cytotoxins. Rev Physiol Biochem Pharmacol 152: 23–47. 15449191 10.1007/s10254-004-0033-5

[13] Qa'Dan M, Ramsey M, Daniel J, Spyres LM, Safiejko-Mroczka B, et al (2002) *Clostridium difficile* toxin B activates dual caspase-dependent and caspase-independent apoptosis in intoxicated cells. Cell Microbiol 4: 425–434. 12102688 10.1046/j.1462-5822.2002.00201.x

[14] Giesemann T, Jank T, Gerhard R, Maier E, Just I, et al (2006) Cholesterol-dependent pore formation of Clostridium difficile toxin A. . J Biol Chem 281: 10808–10815. 16513641 10.1074/jbc.M512720200

[15] Genisyuerek S, Papatheodorou P, Guttenberg G, Schubert R, Benz R, et al (2011) Structural determinants for membrane insertion, pore formation and translocation of Clostridium difficile toxin B. Mol Microbiol 79: 1643–1654. 21231971 10.1111/j.1365-2958.2011.07549.x

[16] Egerer M, Giesemann T, Herrmann C, Aktories K (2009) Autocatalytic processing of *Clostridium difficile* toxin B. Binding of inositol hexakisphosphate. J Biol Chem 284: 3389–3395. 19047051 10.1074/jbc.M806002200

[17] Egerer M, Giesemann T, Jank T, Satchell KJ, Aktories K (2007) Auto-catalytic cleavage of *Clostridium difficile* toxins A and B depends on cysteine protease activity. J Biol Chem 282: 25314–25321. 17591770 10.1074/jbc.M703062200

[18] Reineke J, Tenzer S, Rupnik M, Koschinski A, Hasselmayer O, et al (2007) Autocatalytic cleavage of *Clostridium difficile* toxin B. . Nature 446: 415–419. 17334356 10.1038/nature05622

[19] Yang G, Zhou B, Wang J, He X, Sun X, et al (2008) Expression of recombinant *Clostridium difficile* toxin A and B in *Bacillus megaterium* . BMC Microbiol 8: 192. 18990232 10.1186/1471-2180-8-192 PMC2586027

[20] He X, Sun X, Wang J, Wang X, Zhang Q, et al (2009a) Antibody-enhanced, Fc gamma receptor-mediated endocytosis of *Clostridium difficile* toxin A. Infect Immun 77: 2294–2303. 19307220 10.1128/IAI.01577-08 PMC2687358

[21] Qa'Dan M, Spyres LM, Ballard JD (2000) pH-induced conformational changes in *Clostridium difficile* toxin B. . Infect Immun 68: 2470–2474. 10768933 10.1128/iai.68.5.2470-2474.2000 PMC97448

[22] Lanis JM, Barua S, Ballard JD (2010) Variations in TcdB activity and the hypervirulence of emerging strains of Clostridium difficile. PLoS Pathog 6: e1001061. 20808849 10.1371/journal.ppat.1001061 PMC2924371

[23] Leonard JL (2008) Non-genomic actions of thyroid hormone in brain development. Steroids 73: 1008–1012. 18280526 10.1016/j.steroids.2007.12.016 PMC2601565

[24] Carrington WA, Lynch RM, Moore ED, Isenberg G, Fogarty KE, et al (1995) Superresolution three-dimensional images of fluorescence in cells with minimal light exposure. Science 268: 1483–1487. 7770772 10.1126/science.7770772

[25] Lifshitz Y, Lempert GD, Grossman E (1994) Substantiation of subplantation model for diamondlike film growth by atomic force microscopy. Phys Rev Lett 72: 2753–2756. 10055968 10.1103/PhysRevLett.72.2753

[26] Sun X, He X, Tzipori S, Gerhard R, Feng H (2009) Essential role of the glucosyltransferase activity in Clostridium difficile toxin-induced secretion of TNF-alpha by macrophages. Microb Pathog 46: 298–305. 19324080 10.1016/j.micpath.2009.03.002 PMC2692465

[27] Voth DE, Ballard JD (2005) *Clostridium difficile* toxins: mechanism of action and role in disease. Clin Microbiol Rev 18: 247–263. 15831824 10.1128/CMR.18.2.247-263.2005 PMC1082799

[28] Pruitt RN, Chambers MG, Ng KK, Ohi MD, Lacy DB (2010) Structural organization of the functional domains of *Clostridium difficile* toxins A and B. . Proc Natl Acad Sci U S A 107: 13467–13472. 20624955 10.1073/pnas.1002199107 PMC2922184

